# MalFuzz: Coverage-guided fuzzing on deep learning-based malware classification model

**DOI:** 10.1371/journal.pone.0273804

**Published:** 2022-09-15

**Authors:** Yuying Liu, Pin Yang, Peng Jia, Ziheng He, Hairu Luo

**Affiliations:** School of Cyber Science and Engineering, Sichuan University, Chengdu, SiChuan, China; University of Engineering and Technology Taxila Pakistan, PAKISTAN

## Abstract

With the continuous development of deep learning, more and more domains use deep learning technique to solve key problems. The security issues of deep learning models have also received more and more attention. Nowadays, malware has become a huge security threat in cyberspace. Traditional signature-based malware detection methods are not adaptable to the current large-scale malware detection. Thus many deep learning-based malware detection models are widely used in real malware detection scenarios. Therefore, we need to secure the deep learning-based malware detection models. However, model testing currently focuses on image and natural language processing models. There is no related work to test deep learning-based malware detection models specifically. Therefore, to fill this gap, we propose MalFuzz. MalFuzz uses the idea of coverage-guided fuzzing to test deep learning-based malware detection models. To solve the model state representation problem, MalFuzz uses the first and last layer neuron values to approximately represent the model state. To solve the new coverage calculation problem, MalFuzz uses the fast approximate nearest neighbor algorithm to compute the new coverage. The mutation strategy and seed selection strategy in image model or natural language processing model testing is not appropriate in deep learning-based malware detection model testing. Hence MalFuzz designs the seed selection strategy and seed mutation strategy for malware detection model testing. We performed extensive experiments to demonstrate the effectiveness of MalFuzz. Based on MalConv, Convnet, and CNN 2-d, we compared the modified TensorFuzz and MAB-malware with MalFuzz. Experiment results show that MalFuzz can detect more model classification errors. Likewise, the mutation operation of MalFuzz can retain the original functionality of malware with high probability. Moreover, the seed selection strategy of MalFuzz can help us explore the model state space quickly.

## Introduction

Deep learning has made outstanding achievements in image recognition, automatic driving, natural language processing, malware classification. Therefore, deploying deep learning models to safety- and security-critical domains make it essential to ensure model security. However, massive adversarial attacks on deep learning models [1–6]show that the state-of-the-art models can be easily fooled. Adding an imperceptible perturbation can make model wrongly classified. Thus finding ways to improve the deep learning model security is of great urgency. Similar to improving software security, we must test the software first, then fix the bugs. To enhance the security of the model, the first step is to detect its defects. It calls for an effective approach in model testing. Towards addressing this issue, a variety of proposals have been made for ways to test deep learning models [7–11].

Coverage-guided fuzzing [12] is an effective way to find bugs in software. Therefore, guided by the idea of fuzzing, several works use fuzzing to test deep learning models with promising results. For example, TensorFuzz [13], DLFuzz [14], DeepHunter [15], etc. The idea of the model fuzzing is similar to fuzzing software which aims to trigger more model states. More model states are triggered means the model testing is more adequate. Test cases that cover more model states are better able to find model errors. In addition, Deepxplore [7], DeepGauge [10], DeepCover [11], DeepCruiser [16] and other works have successively proposed test adequacy metrics such as neuron coverage, k-multisection coverage. These works provide coverage metrics for coverage-guided fuzzing in the deep learning model testing.

Malware such as ransomware and Trojan horses pose a huge threat to cyberspace security. Due to the wild growth of novel malware, traditional signature-based methods cannot accurately identify malware. Signature-based methods succeed when the malware was detected and its signature was stored in the anti-virus database. However, once the malware is never spotted, the signature-based approach fails. It calls for effective automatic ways for malware detection and classification. For this reason, there has been an explosion of research papers on malware detection using deep learning [17–20]. Since deep learning-based malware detection models are applied in practical scenarios, it is essential to ensure their reliability. Nevertheless, the above-mentioned deep learning testing techniques are widely studied in domains such as image recognition, while it is rarely applied to malware detection models. Applying testing techniques, like coverage-guided fuzzing to test deep learning-based malware detection models is of great significance.

We have identified two challenges in testing malware detection models. To begin with, testing deep learning models involves mutating the original input. Modifying an image is easy as long as the changes are bounded with a *L*_*p*_-norm, whereas randomly mutating a malware sample can break its format or change its original functionality. Second, considering the whole testing process, the first step is to select a candidate sample from all the samples. If we pick samples with the same probability, we will waste significant testing time on worthless samples. In coverage-guided fuzzing, towards improving testing efficiency, extensive work put forward many approaches for seed selection strategies [21–24]. With regard to testing malware detection models, it is likewise necessary to use a seed selection strategy to improve testing efficiency.

In this work, we address the challenges of testing deep learning-based malware detection models by proposing MalFuzz, a testing framework that applies coverage-guided fuzzing. Malfuzz implements mutation operations that allow malware to preserve its original malicious functionality and executability. We refer to Tensorfuzz [13] in selecting the coverage metrics, which uses the fast approximate nearest neighbor algorithm to calculate the new coverage. However, we believe that the first layer and logits layer of model are equally important in determining whether the model is generating new states, therefore we splice these two layers as indicators for monitoring the model state. Meanwhile, MalFuzz proposes a novel seed selection strategy to boost testing efficiency.

In a nutshell, the following are the major contributions of this paper:

- We apply coverage-guided fuzzing to deep learning-based malware detection model testing. Using coverage to guide the testing process can trigger more model error states, generating more test cases that make the model wrong (i.e., detecting more classification errors in the model). Compared to MAB-malware and TensorFuzz, MalFuzz can explore more model states and generate more test cases within the same amount of time.
- We implement mutation operations that preserve the original malicious functionality and the executability of malware with high probability. Experiment results show that the mutation operations of MalFuzz are effective. The modified TensorFuzz applies random mutation, and it maintains the malware functionality with a probability of about 8.56%. However, the probability of the mutation strategy of MalFuzz maintaining malware functionality is up to 92.9%.
- In order to fuzz a deep learning-based malware detection model more efficiently, we design a seed selection strategy. In order to select a seed to be mutated next, we first determine whether there are seeds in the New-seeds set in the seed pool. If a seed exists in New-seeds, we select a seed from it randomly. If no seeds exist in New-seeds, we will select a seed based on the seed size interval. In tests of Convnet, MalConv, and CNN 2-d, the seed selection approach of MalFuzz outperforms the random selection strategy by 189%, 87%, and 133% in producing new seeds. Likewise, It exceeds the random selection strategy by 115%, 64%, and 95% in generating test cases.
- We implement MalFuzz, a framework to test deep learning-based malware classification models. MalFuzz uses the idea of coverage-guided fuzzing, which uses the first and last layer neuron values to represent the model state and uses the fast approximate nearest neighbor algorithm to compute the new coverage. Meanwhile, it uses effective mutation operations and an efficient seed selection strategy. Experiments show that MalFuzz can effectively test deep-learning-based malware classification models.

## Related work

In this section, we first introduce coverage-guided fuzzing in traditional software testing, discuss how it can explore software inner state and find bugs using coverage as its guidance. Then we discuss some interesting works in deep learning model testing, which is a hotpot in recent years. We will see that it is nature to analogue deep learning model testing with coverage-guided fuzzing. Finally, we review work for adversarial attacks on deep learning-based malware detection models.

### Coverage-guided fuzzing

Fuzzing [12] is one of the most effective methods to find bugs. From the way to produce test cases, there are mutation-based fuzzing and generation-based fuzzing. Mutation-based fuzzing modifies seed inputs to get a new test case(we call this process “mutation”). On the contrary, generation-based fuzzing uses a predefined template to generate test cases. In this paper, we concentrate on mutation-based fuzzing. First, the user (who uses the fuzzer to test a target program) offers a seed file set. A seed file is a target program input. Then the Fuzzer(mutation-based fuzzing framework) construct a seed queue with the seed file set. Next, the Fuzzer continuously mutates the seeds and runs the target program with them. It indicates a bug if the target program crashes.

A program consists of code and data. The state of a program refers to its execution status (what instructions the program has executed and what are the values of the internal variables). Programs pass through many states during their execution. And these states comprise the state space of a program. If a program crashes in one of the execution states, we call this state an error state. Pre-AFL fuzzers are dumb, and they explore program state space blindly. However, AFL guides the fuzzing process with coverage hence allowing exploring the program error state quickly. It significantly improves fuzzing effectiveness. The program state space is extremely large. One input file cannot trigger all states in the program. And we will never be able to detect bugs in untriggered states. Therefore, AFL introduces a coverage metric to indicate that the testing has triggered how many program states. A higher coverage presents sufficient testing for a program. At the same time, a seed that triggers more program states has a higher potential to explore new program states. Hence we should give these seeds more mutation opportunities in the fuzzing process. All the above is the ideology of coverage-guided fuzzing.

However, a completely accurate mathematical definition for the execution states of a program is challenging. Consequently, researchers have proposed several coverage metrics to represent the program states roughly. At the code level [25, 26], there are coverage metrics such as statement coverage, branch coverage, basic block coverage, and data flow coverage. At the model level [27], there are state and transition coverage, etc. Some commonly used coverage metrics are listed as follows:

- The basic block coverage records each basic block that the program passes through during execution (in software engineering, a basic block is the maximum ordered sequence of statements executed in a program). The branch coverage records which conditional branches (if/else statements) get covered. The statement coverage only records which statement gets executed during testing. These metrics focus on code-level coverage and have the advantage of simplicity and ease-of-taking statistics.
- The data-flow coverage focuses on how program variables change from declaration, through assignment, to reference. For variable v, if it is defined at *l*_*i*_ and used at *l*_*j*_, there is an edge between *l*_*i*_ and *l*_*j*_. The data flow coverage tracks variable changes in the code, but it is overly complicated to calculate.
- Software testing is challenging because of its large size. Model-based testing [28] automatically generates test cases by abstracting software behavior models and structural models, thus improving testing efficiency and automation. Model-based coverage calculates how many program behaviors are covered based on software behavior models and is an effective metric for measuring test adequacy.

AFL is a milestone in coverage-guided fuzzing. The previous fuzzers typically use code coverage and basic block coverage as coverage metrics. But these metrics do not contain information about state transitions in program execution. AFL represents the program state with edge coverage, and it inserts instrumentation code into the target program to record the edge coverage information. The effectiveness of coverage-guided fuzzing makes it widely used in testing other types of applications, such as smart contracts [29] and IoT devices [30, 31].

### Deep learning model testing

As mentioned in the previous section, we can use coverage-guided fuzzing to test a wide range of applications. Nowadays, various domains use deep learning to deal with automatic tasks. Hence it is essential to ensure deep learning model security. Therefore, we naturally ask whether proper to introduce fuzzing idea in deep learning model testing.

Obviously, we cannot directly apply software fuzzing to deep learning model testing. First, the software and deep learning models are essentially different. The code directly determines the software execution logic. While the algorithm and training data determine the deep learning model logic. A deep learning model is similar to a function with extensive parameters. Therefore, we cannot use metrics such as basic block coverage to measure explored model states. It is critical to find the appropriate coverage metrics for model testing. Secondly, both testing software and testing models aim to find bugs in the software/model. But the purpose of testing software is to find the code where the bug appears and then fix the bug manually. The purpose of testing the model is to find the samples that make the model wrong (classification errors, translation errors, etc.). Then we fix these errors by retraining.

How to represent the model state? A deep learning model seems like a network with many parameters and computational processes. The input to the model is computed and reaches each network node (usually we call each node a neuron, we also use neuron in this paper). The value of each neuron will change. Different values of all neurons in the network constitute the model state space. But the model state space is infinite, and it is impractical to compute the coverage of endless state space. Therefore, many researchers have proposed metrics that approximately represent model state coverage to fill this gap [7, 10, 32–35]. We list some classic metrics:

- Deepxplore [7] proposes neuron coverage for deep learning model testing for the first time. Experiments demonstrate that using Deepxplore-generated test cases to retrain the model can effectively improve model accuracy. Neuron coverage is analogous to code coverage in software testing. Neuron coverage indicates how many neurons of the target model have an output exceeding a given threshold (code coverage indicates how much of the code in the target program is executed during the test.)
- DeepGauge [10] defines more fine-grained coverage metrics from the neuron-level and the layer-level, respectively. It proposes K-multisection Neuron Coverage, Neuron Boundary Coverage, and Strong Neuron Activation Coverage based on the output values of neurons. Considering each neural network layer, it picks the k neurons with the maximum output value in each layer to define Top-k Neuron Coverage and Top-k Neuron Patterns. The coverage metric proposed by DeepGuage delineates the activation states of neurons more elaborately, and it pays attention to layer-level coverage.
- Deepcruiser [16] aims to test the recurrent neural networks (RNN). RNN and feed-forward DL systems have different network architectures. RNN contains time sequence information. Therefore, it is inappropriate to unroll RNNs into feed-forward neural networks for testing. To fill this gap, Deepcruiser abstracts the RNN into a Markov Decision Process and proposes coverage metrics such as Basic State Coverage and k-Step State Boundary Coverage.

With the emergence of the above coverage, it is natural for many researchers to introduce coverage-guided fuzzing to model testing. DLFuzz [14] is the first differential fuzzing testing framework for deep learning models. It primarily tests image recognition models. The goal of DLFuzz is to maximize the neuron coverage and try to make the model produce classification errors. It models the two as a joint optimization problem and abstracts them into an objective function obj. DLFuzz uses a well-designed selection strategy to choose the neurons to activate in the next iteration. It computes the gradient of obj to the original image *X*_*s*_, treats the gradient as a perturbation added to the original image *X*_*s*_. DLFuzz limits the perturbation to be invisible. Therefore, if the model classifies the original image *X*_*s*_ and the modified image *X*_*m*_ differently, the model has an missification error. DLFuzz adds the modified images that reach new coverages of the model to the seed queue to continue mutating these images. Test4Deep [36] also uses coverage-guided fuzzing, which proposes a more efficient neuron selection strategy to improve neuron coverage. Experiment results show that it generates more test cases and achieves higher coverage than DLFuzz. Tensorfuzz [13] uses coverage-guided fuzzing idea to detect whether the neural network has numerical errors, disagreements between models and their quantized versions, and undesirable behavior in character-level language models. It uses the fast approximate nearest neighbor algorithm to compute new coverages. It computes coverage and determines if the model has errors by extracting coverage and metadata arrays from the Tensorflow graph. TensorFuzz does not only detect model classification errors, it shows that coverage-guided fuzzing can play a significant role in model testing. Robot [37] aims to improve the robustness of the model. They argue that coverage metrics such as neuron coverage are not strongly correlated with model robustness. They, therefore, propose a metric FOL that is more relevant to the robustness of the model. The Robot uses FOL-guided fuzzing to generate test cases for the model and then uses these test cases to retrain the model to improve model robustness.

The aforementioned works use coverage-guided fuzzing to test deep learning models. However, all these works mainly test image and natural language processing models. There is no related work to test deep learning-based malware detection models specifically. Hence our work bridges this gap.

### Adversarial attacks on deep learning-based windows malware classification models

The adversarial attack on the deep learning model adds a small perturbation to the original sample. The perturbation does not change the original properties of the sample. So the model should make the same prediction for the perturbed sample as the original sample. But if the model predicts incorrectly, we call the perturbed sample an adversarial sample.

Currently, many works perform adversarial attacks on malware detection models [38–42]. We mainly survey the adversarial attacks on windows malware detection models. At the 2017 Hacking Conference, Anderson [43] proposed the use of reinforcement learning to attack malware detection models. In previous research work, the modified functions of the malware are likely to be compromised. Therefore, they propose some modification methods for malware, such as adding a function to the import table, changing the section name, and adding a new section. Kolosnjaji [42] perform adversarial attacks on malware classification models that use raw bytes as features (e.g., MalConv). They proposed a gradient-based attack method that adds target bytes to the end of the file byte by byte by calculating the gradient of the target function against the target bytes. Their method maintains the original functionality of the sample and allows the malware to escape successfully. However, this approach takes a long time to compute. The MalConv model, according to Demetrio et al. [44], identifies malware and benign software mainly based on file headers. To mutate the malware, they change the value of bytes in the file header. Demetrio et al. employ a gradient-based approach to compute bytes, similar to the method of kolosniji. However, their approach to changing the file header is more efficient and only needs to change a few bytes. Suciu et al. [38] proposes the Slack FGM method to address the excessive time expense of the gradient-based method of padding bytes at the end of a file. First, they find the slack regions in the file. Then they update these slack byte values by computing the gradient of the loss function over the slack bytes. Similar to the approach of Octavian et al [38]. Kreuk [45] also uses a gradient-based approach to add payload to malware.

Gradient-based approaches are computationally intensive, which is a major disadvantage. Demetrio [46] proposes a series of methods for adversarial attacks on malware detection models. They improve the efficiency of attacks while retaining malware functionality. Their attack injects benign content into the malware (at the end of the file or in a new section). Since benign contents are pre-prepared, hence these operations are highly efficient. In addition, Demetrio has implemented their proposed method and methods proposed by others in python and open-sourced the secml-malware framework [47]. secml-malware is an adversarial attack framework that can attack both white-box and black-box models. It implemented many malware mutation operations (partial dos, full dos, extend, etc.). MAB-Malware [48] generates adversarial samples using reinforcement learning techniques. It models the adversarial attack problem as a multi-armed slot machine problem with a trade-off between getting more rewards and exploring more successful patterns. It employs mutations such as the addition of benign content to the malware and removes unimportant data from the malware. Experiments show that the method can successfully evade popular deep learning-based malware detection models and is effective against some commercial malware detection engines.

The adversarial attack is primarily concerned with manipulating the sample to make it bypass the model than the state of the model. However, model testing focuses on the internal state of the model to measure the adequacy of the test. It extracts the required state information from the model to calculate coverage. Model testing produces test cases that cover the error states of the model. These test cases for re-training the model can improve the robustness of the model [37, 49]. Adversarial attacks on malware detection models can, to some extent, identify model errors. However, model testing produces test cases that cover more of the model error states. It detects model errors more adequately than adversarial attacks.

## The framework of MalFuzz

**Fig 1:**
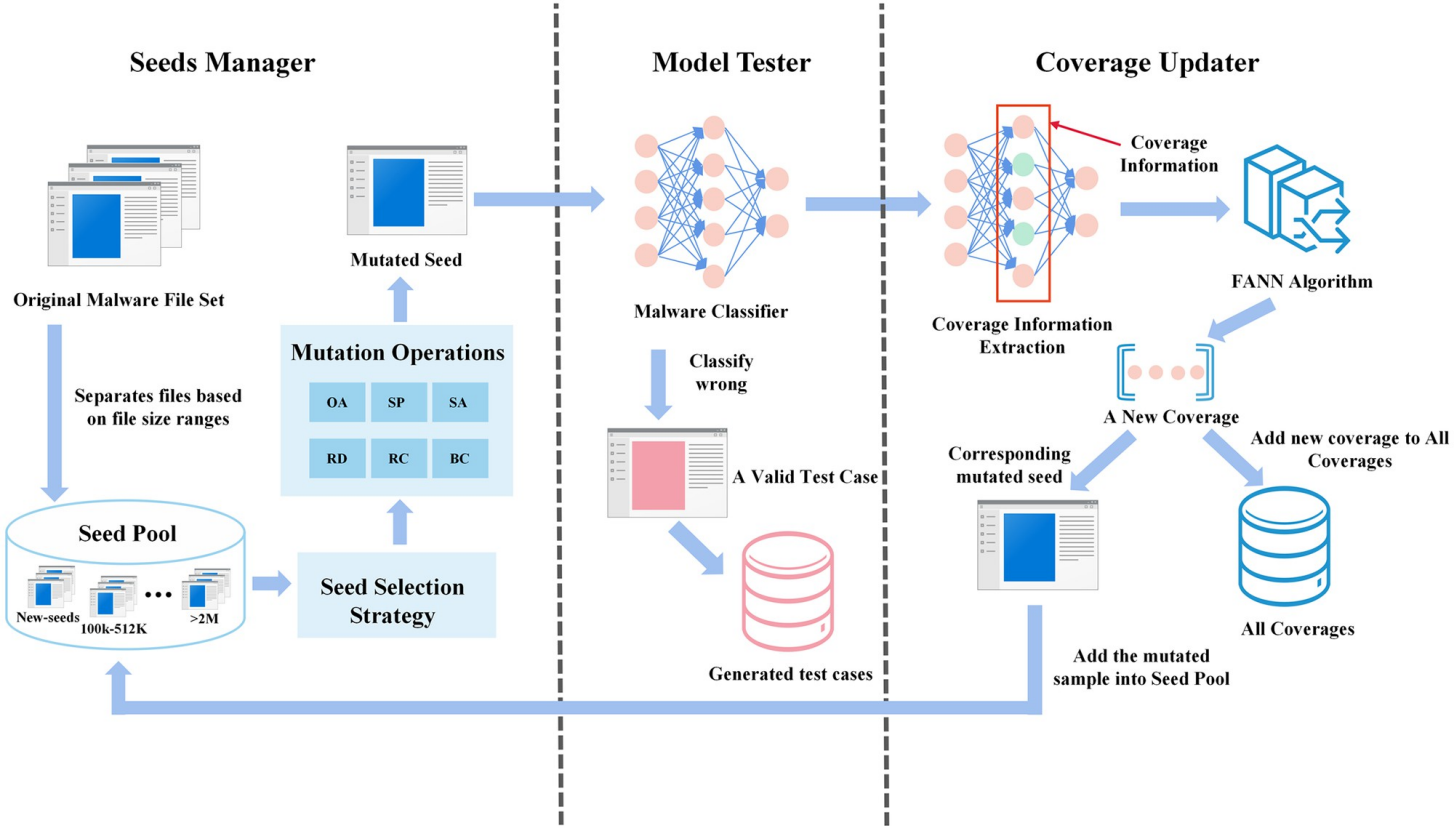
The overall architecture of MalFuzz.

This section describes the overall design and implementation details of MalFuzz. Fig 1 depicts the general architecture of MalFuzz, which consists of the Seeds Manager, the Model Tester, and the Coverage Updater. We go through the internal implementation of these three modules and their linkages in great depth.

To begin with, MalFuzz uses the original malware binary files provided by the user to construct the seed pool and then chooses a seed from the seed pool following its seed selection strategy. Next, MalFuzz selects a mutation operation to mutate the seed. It feeds the mutated seed into the model to acquire prediction, then analyzes whether the model is misclassified and gets coverage information from it. MalFuzz adds the mutated seed to the generated test cases set if the model misclassifies. Finally, MalFuzz calculates whether this mutated seed reaches the new coverage of the model based on the coverage information. MalFuzz adds the modified seed to the seed pool if it triggers a novel coverage. MalFuzz uses coverage as a guide to generate test cases that make the model produce classification errors. MalFuzz stops when all seeds have mutated a specified number of times or when the test process has run through a given number of iterations.

### The Seeds Manager

The Seeds Manager is mainly responsible for seed selection and seed mutation. When MalFuzz starts, the Seeds Manager first constructs a seed pool with the original malware file set provided by the user. During the following test, the Seeds Manager selects a seed from the seed pool using its seed selection strategy and selects a mutation operation to mutate the seed. The Seeds Manager adds the correspondent mutated sample to the seed pool when a new coverage occurs. We mainly introduce seed selection strategies and mutation operations in this subsection.

#### Seed selection strategy

**Fig 2:**
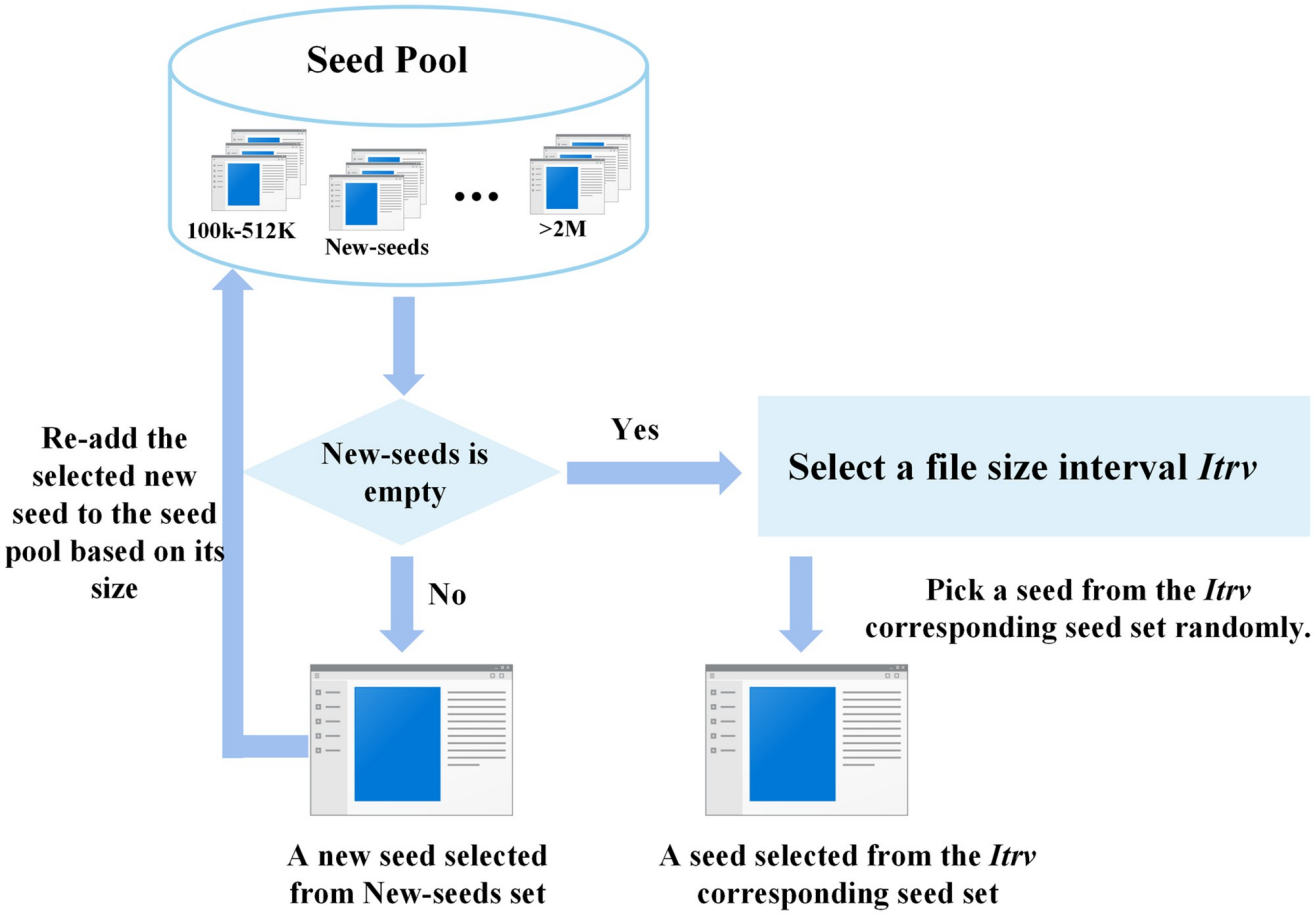
Seed selection strategy.

The state-space of a model is infinite. Intending to detect more error states of the model, we need to find a faster and more efficient way to explore them. During the fuzzing process, many new seeds are produced. At the same time, we keep picking a seed from the seed pool to be mutated. Many seeds are not valuable for exploring the model state space, and it would reduce the efficiency of the test if we consistently select invaluable seeds for mutation. Therefore, we prefer to select seeds that are more potential for detecting model errors and assume these seeds having more mutation potential. To select seeds with high mutation potential, we require an appropriate seed selection strategy. Some malware detection models limit binary file size (.e.g MalConv [50]). We thus assume that the smaller seeds have a promising mutation potential because we can analyze and mutate them more efficiently. According to the idea of traditional software fuzzing, a seed that triggers a new coverage of the malware detection model has a higher mutation potential. Because such seeds can trigger more model error states via further mutations. Taking inspiration from the above two factors, we designed the seed selection strategy for MalFuzz. We show it in Fig 2.

When selecting a seed, MalFuzz first checks for the existence of newly added seeds in the seed pool. The seed pool contains the New-seeds set. When a mutated seed generates a novel coverage, the Coverage Updater adds it to the New-seeds set. If there are seeds in the New-seeds set, the Seeds Manager will choose a seed from the New-seeds set and delete the selected new seed from the New-seeds set. Meanwhile, the Seeds Manager will re-add the selected seed to one of the sets in the seed pool based on seed size interval.

Otherwise, the Seeds Manager selects a seed depending on the seed file size, and it tends to choose smaller seeds. We cannot select seeds strictly by seed size. For instance, if we pick the smallest seed each time, since we won’t delete the seed, MalFuzz will keep selecting it in the test, causing the other seeds to starve. And we suppose to ignore small differences in seed sizes (e.g., a few KB). Therefore, we divide the seeds into different sets by dividing the seed size intervals and give different weights to these intervals. The following Table 1 shows the weight information for the intervals.

**Table 1:** Seed file size intervals and weights.

File Size Interval(B)	Weight
0–100*K*	12
100*K*–512*K*	8
512*K*–1024*K*	6
1*M*–2*M*	4
>2*M*	2

We divide the seed size into five intervals. The probability that the Seed Manager chooses the i-th(i = 0,1,2,3,4) interval is *Prob*_*i*_, which is defined as follows:

$$\begin{array}{c} Prob_{i}=\frac{Weight_{i}}{\sum_{k=0}^{4}Weight_{k}} \end{array}$$ (1)

where *Weight*_*i*_ denotes the weight of the i-th interval. Once we choose an interval, we randomly select a seed from the corresponding seed set.

Using the above seed selection strategy, MalFuzz avoids choosing invaluable seeds blindly and improves model state exploration effectiveness and testing efficiency.

#### Seed mutation strategy

**Fig 3:**
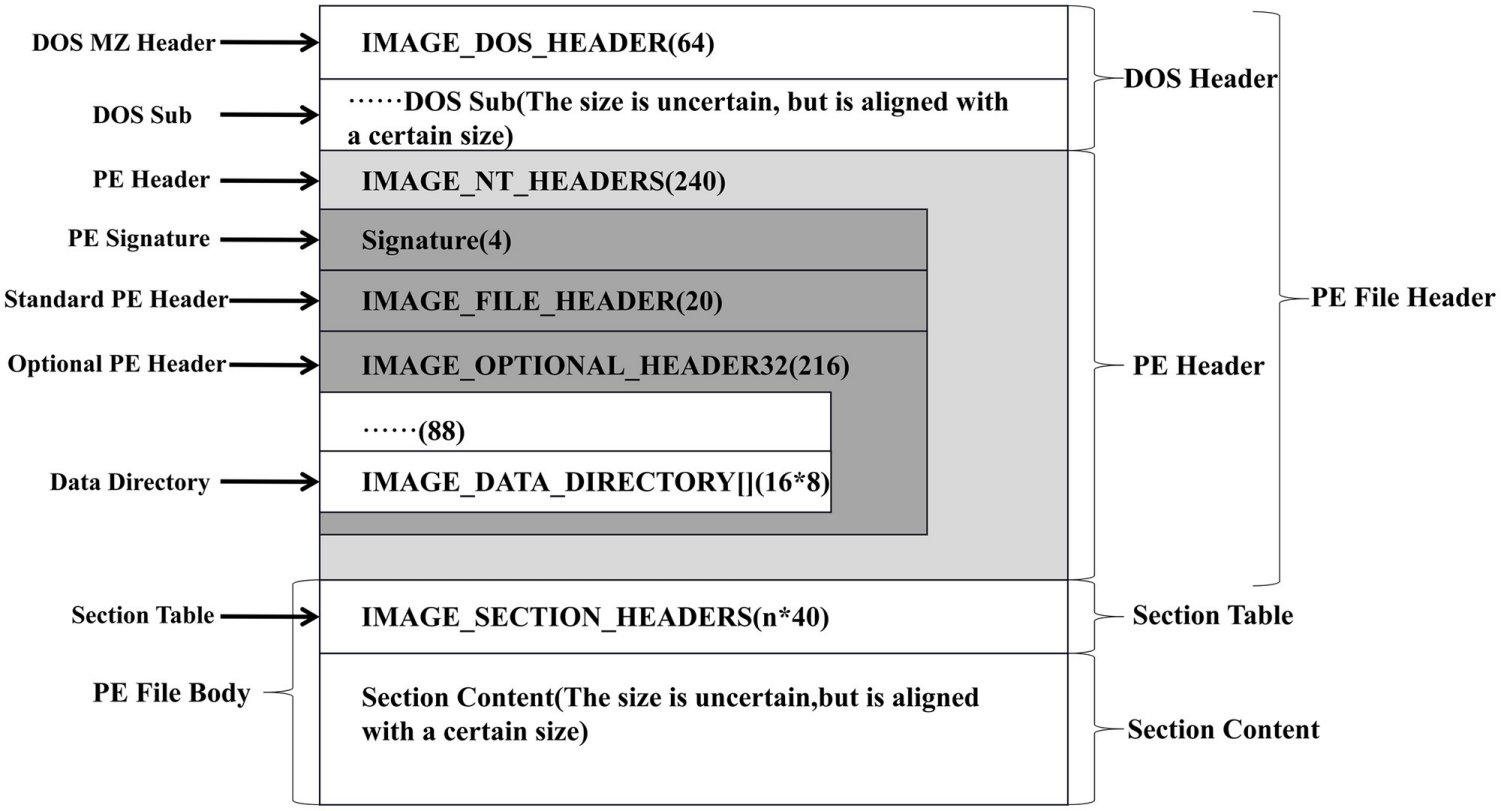
PE file structure.

To increase the diversity of the test cases, we use some mutation operations to mutate the seeds. These mutated samples can explore more model states. The mutation methods used in image or natural language processing model testing are inappropriate for the deep learning-based malware detection model testing. Researchers use adding random noise or gradient-based methods to modify images in testing image classification models. However, the structure of malware is fixed, these methods can corrupt it. The structure of the binary file on the Windows is PE, which is shown in Fig 3.

Binary files store data in a different format in each region. The PE Header store the metadata of the binary file. A section contains information such as code or data. The data organization within each region is also in a different format. We have to analyze the binary file to identify which parts of it we can modify. For instance, if a section has free space in it, we can modify that section. Some information is closely related to whether we can successfully load the program into memory (e.g., PE Signature). Therefore, we cannot modify the information related to the loading of the program.

To modify malware effectively, we have summarized some operations that can alter the binary content of malware without changing its functionality. We show the definition of these operations in Table 2.

**Table 2:** The mutation operations of MalFuzz.

Ops	Name	Description
*OA*	Overlay Append	Appends benign contents at the end of a malware
*SP*	Section Append	Appends random bytes to the free space of a section
*SA*	Section Add	Adds a new section with benign contents
*RD*	Remove Debug	Zero out the debug information in a malware
*RC*	Remove Certificate	Zero out the signed certificate of a malware
*BC*	Break Checksum	Zero out the checksum value in the optional header

We use six operations. RD, RC, and BC clear some execution-independent information from the binaries (the debug information, the certificate, and checksum). OA adds benign content(benign sections that we extracted from clean samples) at the end of the malware. Due to the operating system does not load the contents added at the end of the malware into memory. Hence OA does not affect malicious functions. SP appends random bytes to the free space of a section. Since the program does not reference the contents of the free space during execution, the SP also does not break the malware function. SA adds a new section to the malware and fills it with benign content. Furthermore, SA adds information about the new section to the section table.

Since it does not make sense to do RD, RC, BC multiple times for the same seed file, for RD RC BC, we will only use it once for each seed file. For OA and SA, we randomly select benign content to add to the seeds. We extract massive sections from a large amount of clean sample, thus ensuring that the content added to each seed is as different as possible.

### The Model Tester

During model fuzzing, we need to determine if the model is in error and extract coverage information from the model to calculate new coverage. The Model Tester is responsible for both of these tasks. It first determines whether the model classifies the current mutated seed as malware. If not, then the model has made a classification error. The next question we have to address is what metrics to use to represent the model state.

We analogize software fuzzing to malware detection model fuzzing. For traditional software testing, we use which paths the program has executed to represent the program state and use coverage metrics such as edge coverage to measure test adequacy. For model fuzzing, every combination of each neuron value in a deep learning model constitutes the model state space, but the model state space is infinite. Model fuzzing requires a metric that can represent the model state approximately and is easy to compute. In Tensorfuzz, the authors use the logits layer of the model to compute the new coverage. They claim that the output of the front neurons have a cumulative effect on the output of the neurons in the final layer. However, we believe that it is not appropriate to use only the neuron output of the last layer to represent the model state. The logits layer in some models has a small number of neurons (e. g. Malconv, only 2). As a result, employing merely the logits layer to represent the model state is inadequate. At the same time, the first layer neurons of the model are as important as the last layer neurons. The first layer neurons are the most direct response of the model to the input. Therefore, to solve the model state representation problem, MalFuzz uses the first(except for the input layer) and last layer neuron values to approximately represent the model state.

Therefore, the Model Tester first determines whether the model incorrectly classifies the mutated seed. In case of misclassification, the Model Tester puts the mutated seed into the set of generated test cases. The Model Tester then extracts the first and last layer neuron values from the model as coverage information and passes it to the Coverage Updater. The Coverage Updater then calculates whether this coverage is new.

### The Coverage Updater

For traditional software fuzzing, we need to record coverage information during fuzzing, determine whether the current mutated seed triggers a new coverage, and keep the seeds that trigger a new coverage. Coverage-guided methods help us to detect program defects by exploring the program state space more quickly. Likewise, for model fuzzing, we ought to determine whether the current coverage is a new coverage or not.

MalFuzz represents the model state by splicing neuron values from the first and last layers of the model into a NumPy array. The simple method to compute new coverage is to treat each unique array as a new coverage. However, such coverage calculation approaches do not give valuable guidance for testing malware classification models. This is since the array representing the new coverage might have an endless number of values. As a result, to determine if the present coverage is new, we compute whether it is similar to the coverages generated throughout the test.

**Fig 4:**
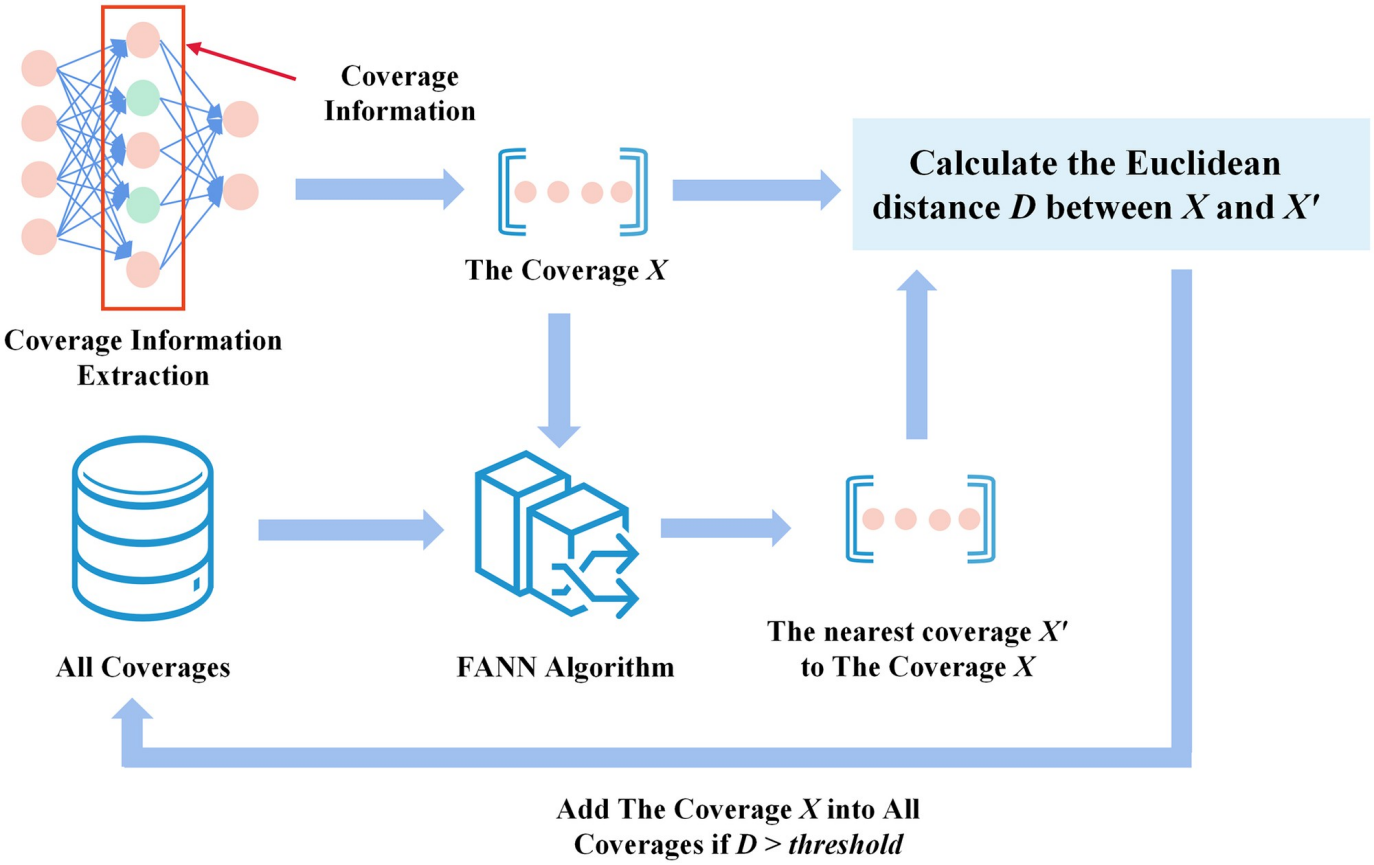
The Coverage Updater.

We show the working process of the Coverage Updater in Fig 4. The Coverage Updater saves all coverage information of the model. We use the fast approximate nearest neighbor algorithm [51] to calculate whether the current coverage is a new coverage. First we use the fast approximate nearest neighbor algorithm to find the nearest coverage to the current coverage among all coverages (using the Euclidean distance to represent the distance between two coverages). Then we see if the Euclidean distance between them is larger than the specified threshold. If the distance between them is greater than the threshold value indicates that the current coverage is new. The Coverage Updater then adds the corresponding mutated seeds to the New-seeds set of the seed pool and adds the current coverage to All Coverages set.

Many third-party open source libraries implement the fast approximate nearest neighbor algorithm. We utilize the sklearn library in the Coverage Updater implementation to discover the approximate nearest neighbors for the current coverage. Users can adjust the threshold dynamically based on the target model.

## Experiments

In this section, we evaluate the efficiency of MalFuzz in testing the deep learning-based malware detection model, as well as in terms of its effectiveness in maintaining the functionality of mutated malware and exploring model state space. Our expperiments aims at answering the following research questions.

- **RQ1**: Is MalFuzz efficient in testing the deep learning-based malware detection model?
- **RQ2**: Can MalFuzz mutate malware effectively?
- **RQ3**: Is the seed selection strategy of MalFuzz effective in explore model state?

### Experiment setup

#### Dataset

From well-known websites (VirusTotal, etc.), we obtained 32,102 benign and malicious samples. Malware comes in many forms, including Trojans, worms, and others. We divided the training set and test set into a 3:1 ratio. To train CNN 2-d, we transformed all malware and benign samples into grayscale images with 128*128 pixels. Table 3 shows the information of the dataset:

**Table 3:** Dataset information for the experiment.

Sample	Training Set	Test Set
*BenignSample*	12000	4051
*MaliciousSample*	12000	4051

#### Setup

We implemented MalFuzz based on TensorFlow 2.2.0, python 3.6, and scikit-learn 0.24.2. The experiments are performed on a workstation equipped with an Intel^®^ Xeon^®^ CPU E5-2680, with 56 CPU and 128 GB of RAM.

To evaluate the effectiveness of MalFuzz, we tested the malware classification model MalConv [50], Convent [52], and CNN 2-d [53]. MalConv uses the raw binary bytes of the binary as features. It first extracts the raw binary bytes from the binary, then performs an embedding operation on the bytes, and finally uses a multilayer neural network to learn the characteristics of malware and benign sample. Convnet also uses raw binary bytes as input to the model. It improves MalConv by increasing the number of convolutional and fully connected layers and using SELU instead of RELU activation functions in the fully connected layers. CNN 2-d uses grayscale images of binary files for malware classification, and it is the model used by Pratikkumar et al. [53] in their paper. Pratikkumar et al. [53] discussed the approach of malware classification using image-based features and deep learning techniques in detail. They also conducted experiments to demonstrate that leveraging image features for malware classification is quite effective.

Individually, we trained MalConv, Convnet, and CNN 2-d. On the test set, MalConv achieved 97% accuracy. Convnet has a 90% accuracy rate. CNN 2-d has a 92% accuracy rate.

As the Original Malware File Set, we gathered 500 malware samples from the Internet. We guaranteed that MalConv, Convnet, and CNN 2-d accurately classified 500 malware. Under this condition, if the model classifies the mutated seeds as benign samples, we consider the model to have generated a misclassification error. To apply OA and SA, we extracted 20000 section contents from benign samples and documented section names and sizes. A seed can be mutated up to 50 times. Otherwise, MalFuzz will ignore it in subsequent testing. The iteration number setting is delicate because MalFuzz cannot explore the model state space adequately if the iteration number is too small. However, MalFuzz will waste test time without improving test effectiveness if the iteration number is too large. We empirically set the iteration number of the experiment to 60,000.

### Is MalFuzz efficient in testing the deep learning-based malware detection model? (RQ1)

We verify the efficientness of MalFuzz and the effectiveness of the mutation strategy of MalFuzz by comparing MalFuzz, MAB-malware, and TensorFuzz. MAB-malware is a state-of-the-art malware detection model adversarial generation tool that uses reinforcement learning to generate adversarial samples. The purpose of MAB-malware is to make all samples escape the model. Therefore, we illustrate the effectiveness of MalFuzz by using test cases generated by MAB-malware to perform adversary attacks on the model and test cases generated by using MalFuzz to test the model. TensorFuzz is a deep learning model testing framework. It primarily tests image and text-based models and modifies images by introducing random noise. TensorFuzz has undergone several changes. The improved TensorFuzz can test malware detection models, but it mutates malware by infusing it with random noise.

**Fig 5:**
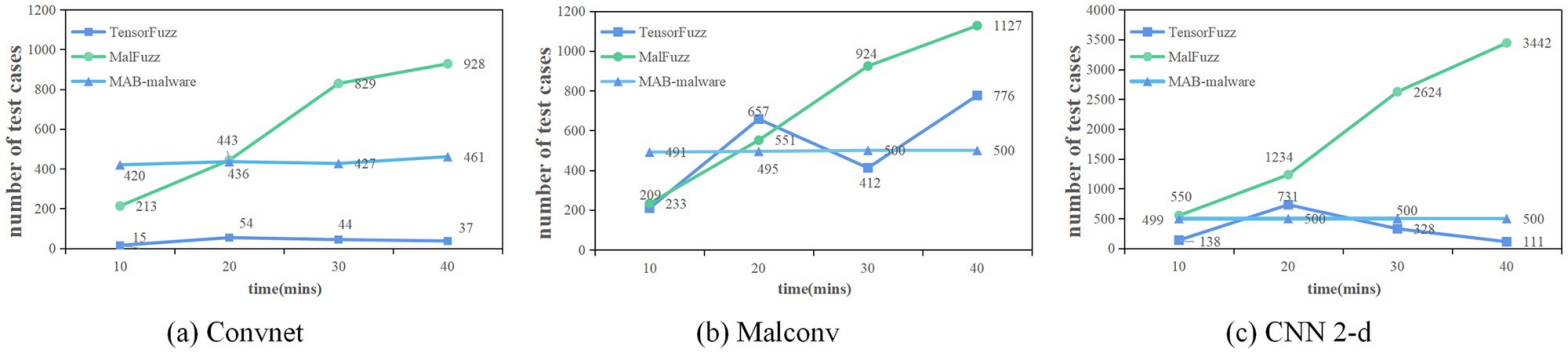
Number of test cases generated in 10,20,30,40 minutes.

To verify the efficiency of MalFuzz, we compare the number of test cases generated by MalFuzz, TensorFuzz, and MAB-malware in 10, 20, 30, and 40 minutes. If a framework generates more test cases in a specified time, it indicates that it can detect model error states efficiently. We use the same Original Malware File Set and the same trained MalConv, Convnet, and CNN 2-d in all three frameworks to ensure a fair comparison. Each experiment runs three times to obtain the average. Fig 5 shows the result.

In the first 30 minutes, MalFuzz generated 829,924,2624 test cases for Convnet, MalConv, and CNN 2-d. TensorFuzz generated 44, 412, and 328 test cases, respectively. MAB-malware generated 427, 500, and 500 test cases. MalFuzz generated far more test cases in the first 30 minutes than MAB-malware and TensorFuzz. As we can see from the figure, after 30 minutes, MalFuzz generated test cases at a slightly lower rate than before (the slope of the folded lines in the graph becomes smaller.). It shows that MalFuzz quickly explored the model state space and detected significant model errors 30 minutes ago. MAB-malware and TensorFuzz generated far fewer test cases than MalFuzz. It suggests that they are not as good at detecting model errors as MalFuzz. Furthermore, we can notice from the figure that the slope of the folded lines of MAB-malware and Tensorfuzz is smaller than that of MalFuzz. Therefore, their ability to probe the model state space is poor than MalFuzz. TensorFuzz is very unstable when testing the model. We can see from the experiment results that the longer the testing time, the fewer classification errors in the model (when testing MalConv, 657 classification errors appeared in 20 minutes, but in 30 minutes, we only found 412 classification errors). MAB-malware, a tool to perform adversarial attacks on the model, aims to make each seed bypass the model, so it generates up to 500 test cases. Moreover, from the experiment results, we can also find that Convnet has fewer errors among these three malware detection models. Compared with MalConv, Convnet has more network layers and can learn more effective malware features. CNN 2-d is the least reliable because the recognition image itself is likely disturbed.

From the experimental results, we can draw the following conclusions. First, MalFuzz enables a quick exploration of the model state space at the beginning of the test. Second, when MalFuzz explores more model state spaces, it can find more model errors. Next, we analyze the reasons for this. MalFuzz saves the mutated seeds that generate new coverage into the seed pool. Furthermore, we can trigger more new coverages with these new seeds. Thus MalFuzz can quickly explore the model state space. When MalFuzz selects seeds, it chooses seeds with high mutation potential. Hence MalFuzz uses these seeds with high mutation potential to detect model errors more efficiently and without wasting testing time. In summary, MalFuzz is efficient in testing the deep learning-based malware detection model.

### Can MalFuzz mutate malware effectively? (RQ2)

Much deep learning model testing frameworks run tests on image-domain models. They alter the images by adding random noise or utilizing gradient-based approaches. However, these mutation methods have the potential to damage the malware structure. Even if they detect model errors, the mutated malware cannot run. As a result, MalFuzz parses binaries and implements mutation operations using the pefile library, and it handles various unexpected cases to prevent destroying the structure of the binaries. For instance, before creating a new section, MalFuzz checks to see if there is adequate space between the last section header and the first section. To prevent compromising some packer functions, we check if there are existing overlay data at the end of the malware before adding overlays to the last section. We parse the file again to see if the parsing was successful. A successful parsing indicates an effective mutation operation.

TensorFuzz represents the model state using the logits layer of the model, and it computes new coverage in the same way as MalFuzz does. However, Tensorfuzz can only add random noise to the malware. MAB-malware has a sophisticated mutation approach as well. Therefore, we compare the number of functionality-preserved test cases generated by MalFuzz, TensorFuzz, and MAB-malware in previous experiments at 10, 20, 30, and 40 minutes to verify the efficacy of the mutation approaches of MalFuzz.

**Fig 6:**
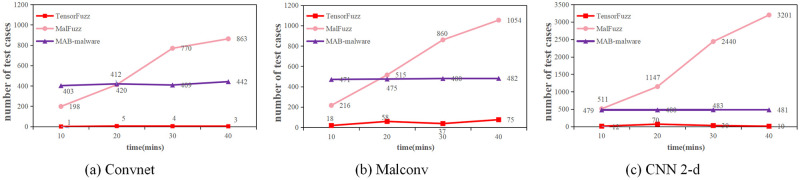
Executable test cases with different mutation strategy.

We use the Cukoo sandbox to check whether the initial and the mutated malware have the same malicious functionality. We compare the API call sequences of the pre-mutation malware with the API call sequences of the post-mutation malware to determine if they have the same functionality and can execute properly. The experimental results are shown in Fig 6.

MalFuzz generated 213, 233, and 550 test cases for Convnet, MalConv and CNN 2-d, respectively, in 10 minutes. The number of test cases with unchanged original functionality is 198,216 and 511. The probability of the mutation strategy of MalFuzz maintaining malware functionality is about 92.9%. MAB-malware generated 420,491,499 test cases separately. The number of test cases with constant functionality is 403,471,479. The probability that MAB-malware keeps the malware functionality is about 96%. However, TensorFuzz generated 15,209,138 test cases for each model individually. The number of executable test cases is 1,18,12. TensorFuzz mutates the malware by adding random noise, and it maintains the malware functionality with a probability of about 8.56%.

We can conclude from the experimental results that TensorFuzz mutates the malware randomly and disrupt the malware structure. In contrast, MalFuzz and MAB-malware use specific mutation operations. These mutation operations modify the binary file by taking into account the binary structure, ensuring that the binary file is function preserved. We analyzed the 15 non-executable test cases generated by MalFuzz for Convnet in 10 minutes. We found that for some packers, adding content to the section area of the packer or at the end of the packer can break the self-extraction process of the packer. However, in general, the mutation operation of MalFuzz is effective. Therefore, MalFuzz can mutate malware effectively.

### Is the seed selection strategy of MalFuzz effective in explore model state? (RQ3)

Coverage-guided fuzzing relies heavily on seed selection. The goal of seed selection is to find seeds with high mutation potential. Following mutation, these seeds are more likely to explore the model state space. To validate the seed selection strategy of MalFuzz, we use random seed selection as a baseline. We compare the number of seed files and the number of test cases generated in 30 minutes using random seed selection and the seed selection strategy of MalFuzz, respectively. The more new seeds generated indicates that the corresponding seed selection strategy is more capable of detecting new states of the model. Similarly, the higher number of test cases indicates that the corresponding seed selection strategy is able to detect more model errors. The experimental setup was the same as the previous experiments. The experimental results are shown in Table 4.

**Table 4:** Comparison of seed selection strategies.

	Test Cases		New Seeds	
Target model	Random Selection	MalFuzz	Random Selection	MalFuzz
Convnet	385	829	73	211
MalConv	561	924	327	614
CNN 2-d	1342	2624	1391	3246

From Table 4, we can see that the seed selection strategy of MalFuzz generateed 829,924,2624 test cases for Convnet, MalConv, and CNN 2-d, respectively, under the same experimental setup. The random selection strategy generated 385,561,1342 test cases, separately. In terms of detecting new coverage of the model, the seed selection strategy of MalFuzz produced 211,614,3246 new seeds for Convnet, MalConv, and CNN 2-d. The random selection strategy produced 153,327,1391 new seeds for these three models. The seed selection approach of MalFuzz outperforms the random selection strategy by 189%, 87%, and 133% in producing new seeds. Likewise, It exceeds the random selection strategy by 115%, 64%, and 95% in generating test cases. The experiment results show that the seed selection strategy of MalFuzz can generate more new seeds and test cases and demonstrate the effectiveness of the MalFuzz seed selection strategy.

We analyze the reasons for the experiment results. Since the model state space is infinite, we use the coverage-guided approach to test the model. However, we should not select the seeds with the same probability. MalFuzz generates new seeds during the testing process. The increase in the number of new seeds indicates that we explore more model state space. The old seeds represent states that we have already detected. In fact, to a large extent, we have detected errors in the detected states. Therefore, we need to focus on detecting errors when the model is in a new state and give a greater selection probability to new seeds. Likewise, as mentioned before, each seed has a different mutation potential, and we should choose seeds with greater mutation potential. For a model like MalConv, it uses the first 2,000,000 bytes of the sample as a feature when detecting malware. For samples smaller than 2,000,000 bytes, it pads the remaining bytes to 255 to meet the model input limit. When testing such models, MalFuzz can inject more benign content into the malware to test whether the model learns enough about the attributes of the malicious and benign samples during training. Meanwhile, MalFuzz is more efficient in analyzing small seeds and can increase testing efficiency. Thus, the seed selection strategy of MalFuzz can balance the exploration of new states of the model and the effectiveness of mutating malware. It can improve the efficiency of testing deep learning-based malware detection models. The seed selection strategy of MalFuzz is effective in explore model state.

## Conclusion

In this paper, we propose the MalFuzz framework for testing deep learning-based malware classification models. MalFuzz uses the idea of coverage-guided fuzzing to detect model classification errors faster by continuously exploring new states of the model. To solve the model state representation problem, MalFuzz uses the first and last layer neuron values to approximately represent the model state. To solve the new coverage calculation problem, MalFuzz uses the fast approximate nearest neighbor algorithm to compute the new coverage. Moreover, MalFuzz designs the seed selection strategy and seed mutation strategy for malware detection model fuzzing specifically. We tested Convnet, MalConv, and CNN 2-d using MalFuzz. The experiment results show that MalFuzz can quickly explore new states of the model and generate more test cases that make the model in error. Likewise, MalFuzz can retain the functionality of the original malware.

However, MalFuzz also has some limitations. The mutation operations of MalFuzz cannot affect advanced features of the malware (e. g. control flow graphs, etc.). We will devise relevant mutation operations in the future to better test models that use these features. The method of calculating new coverage in MalFuzz is too simple, and we will explore metrics that better represent the state of the model and are convenient for calculating model coverage.
